# Nursing staff’s evaluation of facilitators and barriers during implementation of wireless nurse call systems in residential care facilities. A cross-sectional study

**DOI:** 10.1186/s12913-020-4998-9

**Published:** 2020-03-04

**Authors:** Janne Dugstad, Vibeke Sundling, Etty R. Nilsen, Hilde Eide

**Affiliations:** 1 The Science Centre Health and Technology, Faculty of Health and Social Sciences, University of South-Eastern Norway, Drammen, Norway; 2 National Centre for Optics, Vision and Eye Care, Faculty of Health and Social Sciences, University of South-Eastern Norway, Kongsberg, Norway

**Keywords:** Implementation, Nurse call system, Health care technology, Nursing home, Patient safety, Care providers, Workflow, Learning, Determinant framework

## Abstract

****Background**:**

Traditional nurse call systems used in residential care facilities rely on patients to summon assistance for routine or emergency needs. Wireless nurse call systems (WNCS) offer new affordances for persons unable to actively or consciously engage with the system, allowing detection of hazardous situations, prevention and timely treatment, as well as enhanced nurse workflows. This study aimed to explore facilitators and barriers of implementation of WNCSs in residential care facilities.

****Methods**:**

The study had a cross-sectional descriptive design. We collected data from care providers (*n* = 98) based on the Measurement Instrument for Determinants of Innovation (MIDI) framework in five Norwegian residential care facilities during the first year of WNCS implementation. The self-reporting MIDI questionnaire was adapted to the contexts. Descriptive statistics were used to explore participant characteristics and MIDI item and determinant scores. MIDI items to which ≥20% of participants disagreed/totally disagreed were regarded as barriers and items to which ≥80% of participants agreed/totally agreed were regarded as facilitators for implementation.

****Results**:**

More facilitators (*n* = 22) than barriers (*n* = 6) were identified. The greatest facilitators, reported by 98% of the care providers, were the expected outcomes: the importance and probability of achieving prompt call responses and increased safety, and the normative belief of unit managers. During the implementation process, 87% became familiar with the systems, and 86 and 90%, respectively regarded themselves and their colleagues as competent users of the WNCS. The most salient barriers, reported by 37%, were their lack of prior knowledge and that they found the WNCS difficult to learn. No features of the technology were identified as barriers.

****Conclusions**:**

Overall, the care providers gave a positive evaluation of the WNCS implementation. The barriers to implementation were addressed by training and practicing technological skills, facilitated by the influence and support by the manager and the colleagues within the residential care unit. WNCSs offer a range of advanced applications and services, and further research is needed as more WNCS functionalities are implemented into residential care services.

## Background

Traditional nurse call systems (NCSs) used in residential care facilities rely on patients to summon assistance for routine or emergency needs. NCSs are light call-, care communication-, call-, paging- or patient call systems, and may constitute of a variety of features supporting the main nurse call function, which is to support patient safety and facilitate communication between the patient and the nursing staff. NCSs are well accepted health technologies [1], integrated in standards (e.g. the German DIN VDE 0834 standard) and recommendations (e.g. the British Health Technology Memorandum 08–03; the Norwegian State Housing Bank recommendation HB 8.C.8.) to ensure that the healthcare organizations apply statutory requirements. The importance of implementing a multifunctional NCS that addresses the users’ needs and supports effective communication between patients, healthcare providers and management, is emphasized [2]. Research on NCS has predominately been hospital-based and has primarily reflected the major driving forces of technical development, namely to enhance patient safety through reduced call response time and to eliminate alarm fatigue among healthcare providers [2–5]. In the residential care sector, research on alarm fatigue and corresponding patient safety issues has been related to monitoring systems [6–9]. As a part of the digital transformation of healthcare services and based on a recommendation from the Norwegian Directorate of Health in 2017, wireless nurse call systems (WNCSs) are currently implemented in Norwegian residential care facilities (RCFs). Monitoring technologies and smart technologies have primarily been implemented as stand-alone solutions, but are now increasingly integrated in WNCSs and this integration represents a novelty in residential care settings. Therefore, there is a need to investigate the implementation of WNCSs in RCFs.

### New affordances for residents

Traditional NCSs consist of bedside call buttons or cords, light domes and audible alarms in corridors and at the nurses’ stations, and pagers or portable phones carried by nursing staff. These systems rely on the patients’ capability to assess their own condition and summon assistance from healthcare providers for routine or emergency needs, thereby leaving the patients with some control in the care situation [10–12]. However, capabilities as well as needs of older people living in RCFs are increasingly complicated by serious illnesses, dementia and comorbid conditions [13]. To remediate this, pervasive and internet-of-things (IOT) technologies in WNCSs model the input from mobile transceivers (wristbands or pendants) and ambient or body-worn sensors by ontologies or statistics, spatiotemporal reasoning and decision-making techniques. The WNCSs allow detection of unattended events and hazardous situations, support prevention and timely treatment, and reduce injury and harm [14–17]. Thus, WNCSs offer new affordances, properties and interactions, to patients who are not able to actively or consciously interact with the systems. Compared to fixed appliances within limited spaces as provided by traditional, wired systems [18], WNCSs also offer increased mobility for all users.

### Potential for improved alarm management

Nursing staff is responsible for handling calls [11] and rely on the NCS to coordinate their work [19]. The NCSs generate data on the number of calls and response time, which enables the management to monitor the performance [20]. Alarm adverse events have been found to involve human, organizational and technical factors [3]. The most salient is alarm fatigue, i.e. healthcare providers’ increased response time and decreased response rate to alarms [21–23]. Thus, clinical alarm systems are rated as one of the most salient health technology hazards, imposing risk to patient safety [24]. The increased number of appliances integrated in the WNCSs could potentially add to the number of alarms constantly interrupting the nursing staff’s work, and compromise the caring relationship with patients [1, 10, 18, 19, 25]. Converged mobile technology addresses these challenges and adds affordances to the nursing staff’s utilization of the systems [26]. WNCSs integrate middleware technology combined with smart-phones and have been found to successfully filter and bundle clinically significant alarms, resulting in real-time alerts and escalations for urgent alarms while at the same time reducing the number of redundant alarms [27]. In addition, safer and more efficient workflows are allowed by algorithms for smart routing of patients’ requests between care providers [28].

### Implementation of transformative digital health technologies

Full-scale, transformative implementation processes are expected due to the wide-spread use of the WNCSs by nearly all patients and nursing staff within a healthcare facility [29, 30], and due to the range of new functionalities offered by the WNCSs compared to traditional call systems, as detailed above. In two longitudinal case studies, we have explored the implementation processes of novel, digital monitoring technology in Norwegian RCFs. The implementation processes were complex, time-consuming and represented radical innovation [31], and resistance to technology and implementation strategies emerged as an immediate phenomenon [32]. Since these studies were undertaken, the municipal sector had gained experience from pilot-implementations, and a national strategy of WNCS implementation with improved technological systems had been introduced. There was a need to explore how the care providers experienced the implementation of WNCS.

Residential care facilities, long-term care settings and nursing homes are characterized by a large portion of unskilled or semi-skilled staff, authoritative, hierarchical (top-down) communication and represent complex settings [33]. This complexity should be accounted for and attended to in order to facilitate successful implementation, and develop new knowledge and practice. Within this complexity, factors that affect implementation processes include the physical environment and infrastructure, availability of time and resources, availability of staff training, availability of support, receptiveness of organizational culture, involvement of all stakeholders, demonstrable benefits of the change and empowering leadership [34–39].

Implementation strategies are “methods or techniques used to enhance the adoption, implementation, and sustainability of a clinical program or practice” [40]. A recent study of the relationship between barriers and implementation strategies concluded that detailed evaluations are needed [41]. Theoretical determinant frameworks can be applied to explore how human, organizational, technical and other contextual factors or implementation strategies affect the implementation processes [42]. In this study, we use the Measurement Instrument for Determinants of Innovations (MIDI) framework [43–45] to evaluate facilitators and barriers of implementation of WNCSs in residential care settings.

To the best of our knowledge, facilitators and barriers related to human, organizational or technical factors, as well as strategies included in full-scale implementation of WNCSs, have not been described in the literature. This paper aims to explore healthcare providers’ evaluation of facilitators and barriers during implementation of WNCSs in residential care settings.

## Methods

### Design and study setting

The study had a cross-sectional design. The sample population of care providers included registered nurses, healthcare workers (a registered healthcare profession in Norway with a certificate of apprenticeship) and other health professions (physiotherapists, learning disability nurses, and nutritionists), in five RCFs in South-Eastern Norway. The RCFs were actively engaged in full-scale implementations of WNCSs when the study was undertaken. All units (*n* = 16) involved in the implementation of WNCS at the time of the study were included. The RCFs offered round-the-clock services, and consisted of units or wards providing a variety of housing options, Table 1. The residents were primarily older persons suffering from multi-morbidity and mild to severe cognitive deficiencies. At least one long-term somatic care unit was included for each RCF. Moreover, five secluded units with care services accustomed to the needs of persons with moderate to severe dementia were included.

**Table 1:** Characteristics of the residential care facilities

	RCF1	RCF2	RCF3	RCF4	RCF5
RCF location	Rural	Suburban	Suburban	Suburban	Urban
Somatic unit profiles	Short-term rehabilitation unit; acute care unit; nursing home units; long-term sheltered housing units	Long-term sheltered housing units	Nursing home units	Nursing home units; long-term sheltered housing unit*	Nursing home unit
Units included	8	2	2	1	3
Somatic units	5	2	2	1	1
Secluded dementia units	3	0	0	0	2
Residents	136	46	50	26	39
Healthcare professionals^	190	49	50	23	34

Abbreviations: *RCF* residential care facility. *Only the long-term somatic sheltered housing unit with round-the-clock services was included in the study. ^The number of employees eligible to participate in the survey (nurses, healthcare workers or other healthcare professions)

### Data collection

The data collection was based on a questionnaire (Additional file 1), and took place between September 2017 and February 2019. It was performed sequentially according to recruitment of RCFs, within their first year of WNCS implementation. We identified and approached the managers of RCFs that recently had procured WNCSs, of whom all consented for the RCF to take part in the study. The care providers were informed about the survey in nursing staff meetings by the researchers in RCF2–5. Care providers of RCF2–5 received written information about the study, the informed consent form, a paper questionnaire, and a return envelope, and were asked to complete the questionnaire. A digital survey was developed on demand by the RCF1 management. The digital survey was provided by and administered via the University of Oslo’s research survey platform Nettskjema. We piloted the digital survey through several iterations within a group of approximately 10 laypersons, care providers, and researchers aged 18–70, in order to ensure usability in the smart phone format. After the initial information meeting for the care providers in RCF1, which was conducted by the manager, a text message containing information about the study, a request to fill out the questionnaire, and a link to the web-based survey was sent to the care providers’ private mobile phones through the RCF administrative system. Care providers in all RCFs received a friendly reminder after one week.

### The measurement instrument for determinants of innovations (MIDI)

MIDI is a theory- and evidence based questionnaire which is suitable for research, as well as for practical implementations [44]. Departing from the diffusion of innovations theory [46], the development of MIDI was informed by a systematic literature review of empirical studies, and refined through Delphi studies as well as eight empirical studies of the implementation of evidence-based innovations [43, 44]. MIDI was designed to improve the understanding of how critical determinants affect implementation of innovations within larger healthcare organizations, allowing a more precise targeting of the innovation strategies applied [44]. MIDI captures 29 determinants (D1–29) in four categories to be evaluated by care providers who are adopting the innovation [45]. The first category is related to the innovation (D1–7), the second to the adopting user (D8–18), the third to the organization (D19–28), and the fourth category to the socio-political context (D29). With respect to instrument reliability, Kuunders, Jacobs, Goor, Bon-Martens, Oers, and Paulussen [47] have reported good internal consistency, Cronbach’s coefficient α score ranged from 0.61 to 0.90 for the MIDI determinants. We found excellent internal consistency, Cronbach’s coefficient α = .90 (total scale), α = .76 (the innovation scale), α = .84 (the adopting user scale) and α = .84 (organization scale; including items with Likert response scale only).

### Adoption of MIDI to the WNCS implementations

The questionnaire consisted of an initial section for participants’ background information, such as gender, age, profession and years of work experience, followed by MIDI adapted to the WNCS implementations [44]. In order to adopt the questionnaire to the implementation processes in the RCF contexts, information about the systems, the new routines and responsibilities related to the systems, as well as the implementation strategies applied, was collected in accordance with a procedure recommended by Dugstad, Sundling, Nilsen and Eide [48]. For each of the implementations, information was collected in meetings with a representative from the municipal healthcare top management, the RCF unit management team, the municipal IT support service, and the vendors. We co-created an adapted questionnaire with a WNCS super user. Finally, the questionnaire was quality-assured item-by-item in meetings with the respective RCF managements. An overview of the determinants, number of items and response scales is presented in Table 2, and the 85 items are further detailed in Additional files 1 and 2. Each item of the MIDI questionnaire is scored on a scale from 1 to 3, 1 to 4, 1 to 5, or 1 to 7, Table 2, according to the MIDI manual [45]. The manual suggested dichotomous scales (yes/no) for D18, D25 and D26. However, we also included a third response option “I don’t know” based on advice provided in the preparatory meetings with the RCFs.

**Table 2:** Overview of MIDI determinants, number of items, and response scales

Determinant	No of items	Scale
Innovation: WNCS		
D1 Procedural clarity ^	1	1–5
D2 Correctness ^	1	1–5
D3 Completeness ^	1	1–5
D4 Complexity *	1	5–1
D5 Compatibility ^	1	1–5
D6 Observability ^	1	1–5
D7 Relevance for resident ^	1	1–5
Adopting user: care provider		
D8 Personal benefit ^	7	1–5
D8 Personal drawback *	1	5–1
D9 Outcome expectation, importance ^	3	1–5
D9 Outcome expectation, probability ~	3	1–5
D10 Professional obligation ^	1	1–5
D11 Resident/family satisfaction ^	2	1–5
D12 Resident/family cooperation ^	2	1–5
D13 Social support ^	8	1–5
D14 Descriptive norm **	1	1–7
D15a Normative beliefs ~	10	1–5
D15b Motivation to comply ^#^	10	1–5
D16 Self-efficacy ~	10	1–5
D17 Knowledge ^	12	1–5
D18 Awareness of content ¤	1	1–4
Organization: residential care facility unit		
D19 Formal ratification ¨	1	1–3
D20 Staff turnover ^	1	1–5
D21 Staff capacity ^	1	1–5
D22 Financial resources ^	1	1–5
D23 Time available ^	1	1–5
D24 Material resources ^	1	1–5
D25 Coordinator ¨	2	1–3
D26 Unsettled organization ¨	1	1–3
D27 Information available ^	1	1–5
D28 Performances feedback ^	1	1–5
Socio-political context: Norwegian legislation		
D29 Legislation and regulations ^	1	1–5

Abbreviations: *MIDI* Measurement Instrument for Determinants of Innovation, *WNCS* wireless nurse call system, *D* determinant. Response scales: ^ 1; totally disagree, 2; disagree, 3; neither agree nor disagree, 4; agree, 5; totally agree. * 1; totally agree, 2; agree, 3; neither agree nor disagree, 4; disagree, 5; totally disagree. ~ 1; most definitely not, 2; definitely not, 3; perhaps, perhaps not, 4; definitely, 5; most definitely. ** 1; not a single colleague, 2; almost no colleagues, 3; a minority, 4; half, 5; a majority, 6; almost all colleagues, 7; all colleagues. # 1; very little, 2; little, 3; not a little, not a lot, 4; a lot, 5; a great deal. ¤ 1; I’m not familiar with the WNCS, 2; I’m familiar with the WNCS, but have not explored it, 3; I’m familiar with the WNCS and have some experience with it, 4; I’m well acquainted with and use the WNCS. ¨ 1; no, 2; yes, 3; I don’t know

### Statistical analysis

The Statistical Package for Social Sciences 21.0 was used for data analysis. In line with Verberne, Kars, Schepers, Schouten-van Meeteren, Grootenhuis, and van Delden [49], we defined MIDI items to which ≥20% of participants responded ‘totally disagree/disagree’ as barriers and items to which ≥80% of participants responded ‘agree/totally agree’ as facilitators for implementing the WNCS. Descriptive statistics (mean, standard deviation, median, range and percentage) were applied to the participant characteristics and MIDI scores. As the MIDI scale is not ordinal, the Kruskal Wallis test was used to explore differences between groups, with *p*-value ≤ .05 considered statistically significant. The internal consistency of the MIDI questionnaire, was assessed by Cronbach’s coefficient α [50].

### Research ethics

The research was done in line with the Helsinki Declaration [51]. The Norwegian Data Service for Social Sciences acts as the University of South-Eastern Norway’s institutional review board and approved the project according to the Personal Data Act (approval no. 918960). The participants received information about the study both orally and in writing, and provided informed consent in writing or by responding to the digital questionnaire.

## Results

### The WNCS implementations

The WNCSs were based on digital platform solutions with a multitude of integrated technological applications and features, Table 3. The implementation of the WNCSs had a stepwise approach in all RCFs, starting with basic alarm and monitoring functions compatible with existing workflows. New features would be included after the initial phase of implementation. The transition to the novel WNCSs relied on investments, approved and coordinated on higher organizational levels. The municipal administrations governed both the healthcare service organizations responsible for the RCFs and the information technology (IT) service organizations responsible for the support of the WNCSs. Further, they were in charge of the procurement of the WNCSs and the long-term service and support agreements with the vendors. Implementation coordination teams headed by the RCF managers were in charge of the implementations. All the municipalities included a digital transformation facilitator in their coordination team. The RCF management, including the unit managers, were responsible for the implementation strategies on a daily basis, Table 4.

**Table 3:** Characteristics of the WNCSs

WNCS device/application	Features	RCF				
		1	2	3	4	5
Mobile transceiver for resident	Wrist band or pendant	x	x	x	x	x
	Call function	x	x	x	x	x
	Fall detection (wearable based)					
	Indoor localization			x	*	x
	Outdoor tracking (e.g. GPS)	*		x	*	x
	Speech interface			x	x	x
	Programmed to unlock/open/lock doors	x				
Alarm button (bedside/bathroom)	Optional for residents with dementia	x			x	
Ambient sensors for resident monitoring	Bed-exit sensors	x	x	x	x	x
	Door sensors (indoor)	x			x	
	Front-door sensors	x		x	x	x
	Fall detection (ambient or vision based)					
	PIR based motion detection	x	x	x	x	x
Smartphone for care providers	Receive and mange calls/alerts from residents	x	x	x	x	x
	Receive and mange alerts from ambient sensors	x	x	x	x	x
	Call on /talk to colleagues	x	x	x	x	x
	Emergency call function		x	x	x	x
	Task assignment and prioritizing	x	x	x	x	x
Wall mounted modules for care providers in corridors/common rooms	Alarm button	x	x	x		x
	Means to sign out alarms	x				
	Speech interface		x	x		x
	Emergency call function	x				
Wireless nurse call system solution	Adjustable, individual settings for residents	x	x	x	x	x
	Managerial reports of system access by whom and when, number of alarms, response times, missed alarms	x	x	x	x	x
	Smart network access points	x	x	x	x	x
	Alarm back-up system if wifi fails	x		x		x
	Battery back-up if power outage	x	x	x	x	x
	Integrated with fire alarm system	x		x	x	x

Abbreviations: *WNCS* wireless nurse call system, *RCF* Residential care facility, *GPS* global positioning system, *PIR*; Passive infra-red. X = Implemented. *Functionality is offered by the system and will be integrated into the care service at a later stage

**Table 4:**
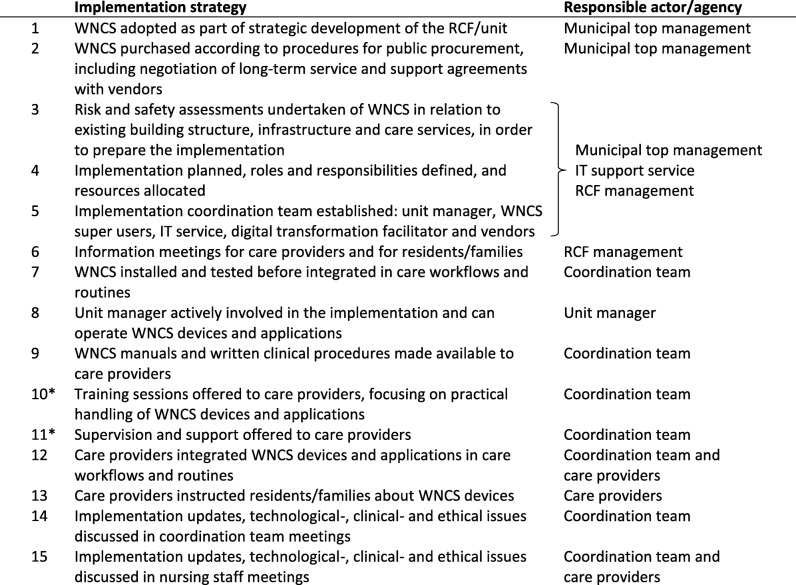
Implementation strategies related to WNCS

Abbreviations: *WNCS* wireless nurse call system, *RCF* residential care facility. * Implementation strategies not adopted by RCF1

The management of RCF1 approached the WNCS implementation as a regular update of the previous NCS, and did not appoint WNCS super users or offer any training to the care providers. In contrast, the other RCF managements approached the WNCS implementations as digital transformative processes, and adopted implementation strategies to provide training, supervision and support to the care providers. The WNCS super users received extensive training and supported their colleagues in the use of the WNCS.

### Participant characteristics

In total, responses from 98 care providers were analysed in the study, Table 5. The total response rate was 28.3%, lowest (10.5%) for the digital survey and ranging from 44.2 to 52.2% for the paper-based survey.

**Table 5:** The response rates and participants’ professional background by RCF, n(%)

Professional groups Response rate (n,%)	Total 98 (28.3)	RCF1 20 (10.5)	RCF2 25 (51.0)	RCF3 26 (52.0)	RCF4 12 (52.2)	RCF5 15 (44.2)
Healthcare workers	61 (62.2)	16 (80.0)	16 (64.0)	15 (57.6)	4 (33.3)	10 (66.7)
Nurses	26 (26.5)	3 (5.0)	5 (20.0)	9 (34.6)	6 (50.0)	3 (20.0)
Other health professions	11 (11.2)	1 (0.5)	4 (16.0)	2 (7.6)	2 (16.7)	2 (13,3)
Super users*	19 (19.4)	0 (0.0)	4 (16.0)	8 (30.8)	2 (16.7)	5 (0.33)

Abbreviations: *RCF* residential care facility. *The super users were nurses (*n* = 7), healthcare workers (*n* = 10) and other healthcare professions (n = 2)

The care providers’ average age was 43.1 years (range 21–69; *n* = 95). The mean duration of professional work experience was 16.6 years (range 0–50; *n* = 94) and the mean duration at current job was 8.0 years (rage 0–32; *n *= 95). There were no differences in the professional groups between the RCFs. However, there were statistically significant demographic differences between the professional groups, Table 6.

**Table 6:** Participants’ gender, age and years of experience by profession, n(%)

	Healthcare workers	Nurses	Other health professions
Female (*n* = 83)	51 (61.4)	22 (26.5)	10 (12.0)
Male (*n* = 14)	10 (71.4)	3 (21.4)	1 (7.1)
Mean age (yr; range) *	46.1 (21–69)^	38.4 (22–66)~	37.6 (22–54)
Mean duration of professional experience (yr; range) **	18.7 (0–50)^	14.6 (0.5–40)~	10.3 (2.5–30)
Mean duration at current job (yr; range) ***	10 (0–32) ^	5.5 (0.5–29)	3.5 (0.5–9)

Statistically significant difference between professional groups, Kruskal Wallis test *p = .011, **p = .042 and ***p = .028. Missing data for ^ 3 participants for duration of professional experience and mean duration of current job and ~ 1 participant for duration of professional experience

### MIDI scores, facilitators and barriers

Overall, the care providers gave a positive evaluation of the WNCS implementation; MIDI scores of all the 85 questionnaire items were either neutral or positive to the innovation. The care providers identified a number of facilitators and barriers that will be presented in the following. We report the proportion (%) of the care providers who have responded ‘agree/totally agree’ regarding facilitators or ‘totally disagree/disagree’ regarding barriers, for each determinant or item. Determinants and items not identified as facilitators or barriers are detailed in Additional file 2.

#### Facilitators

The item to which ≥80% of responding care providers responded ‘agree/totally agree’ or corresponding values in the most positive end of the response scale, are presented as facilitators in Table 7. The facilitators with the highest scores, to which 98% of all the care providers (*n* = 98) definitely or most definitely agreed, were the normative belief of the manager (D15A) and that the WNCS probably increased the safety for residents, and probably increased the feeling of safety for families (D9). Further, 95% expected that the WNCS probably would provide faster assistance to the residents (D9), and 98% of the care providers found it important that the WNCS increased the safety for residents, provided faster assistance to residents, and increased the feeling of safety for families.

**Table 7:** Facilitators for WNCS implementation

Determinant/*item*	Mean	SD	Median	Range	N	TD/D (%)	A/TA (%)
D9 Outcome expectation							
*a) It is important that WNCS increases safety for residents*^	3.78	(0.47)	4	(2–4)	98	0	98
*b) It is probable that WNCS increases safety for residents*~	4.63	(0.61)	5	(1–5)	98	1	98
*c) It is important that WNCS gives faster assistance to residents*^	3.76	(0.45)	4	(2–4)	97	0	98
*d) It is probable that WNCS gives faster assistance to residents*~	4.54	(0.74)	5	(1–5)	98	3	95
*e) It is important that WNCS increases safety for families*^	3.67	(0.51)	4	(2–4)	98	0	98
*f) It is probable that WNCS increases safety for families*~	4.56	(0.54)	5	(3–5)	98	0	98
D10 Professional obligation^	4.02	(0.91)	4	(1–5)	98	7	84
D13 Social support							
*a) To use WNCS, I can get support from the manager*^	4.23	(0.97)	4.5	(1–5)	96	6	80
*d) To use WNCS, I can get support from a nurse*^	4.18	(0.81)	4	(1–5)	95	2	80
D14 Descriptive norm**	5.99	(1.05)	6	(3–7)	96	5	90
D15A Normative beliefs							
*a) The manager expects me to use WNCS*~	4.76	(0.48)	5	(3–50	98	0	98
*d) A nurse colleague expects me to use WNCS*~	4.55	(0.66)	5	(2–5)	97	1	92
*e) A healthcare worker colleague expects me to use WNCS*~	4.48	(0.65)	5	(2–5)	97	1	93
D15B Motivation to comply							
*a) I comply with opinions of the manager*#	4.52	(0.68)	5	(2–5)	96	2	92
*d) I comply with opinions of a nurse colleague*#	4.42	(0.59)	4	(3–5)	95	0	91
*e) I comply with opinions of a healthcare worker colleague*#	4.40	(0.57)	4	(3–5)	94	0	92
D16 Self-efficacy							
*d) I can receive an alarm on the smart phone*~	4.56	(0.58)	5	(3–5)	98	0	96
*e) I can manage an alarm on the smart phone*~	4.49	(0.69)	5	(2–5)	97	1	90
*f) I can use the emergency call application to alert a colleague*~	4.46	(0.79)	5	(1–5)	98	3	91
*h) I can provide feed-back to the manager or super user*~	4.31	(0.87)	5	(1–5)	97	3	82
D17 Knowledge							
*a) I know enough to use WNCS*^	4.18	(0.77)	4	(1–5)	97	3	86
D18 Awareness of content of the WNCS¤	3.25	(0.75)	3	(1–4)	97	12	86

Abbreviations: *WNCS* wireless nurse call system, *TD/D* totally disagree/disagree, *A/TA* agree/totally agree. A/TA value ≥80% indicates that the determinant or item is a facilitator for the implementation. The percentage values are rounded to the nearest whole number. Response scales: ^1; totally disagree, 2; disagree, 3; neither agree nor disagree, 4; agree, 5; totally agree. ~ 1; most definitely not, 2; definitely not, 3; perhaps, perhaps not, 4; definitely, 5; most definitely. ** 1; not a single colleague, 2; almost no colleagues, 3; a minority, 4; half, 5; a majority, 6; almost all colleagues, 7; all colleagues. # 1; very little, 2; little, 3; not a little, not a lot, 4; a lot, 5; a great deal. ¤ 1; I’m not familiar with the WNCS, 2; I’m familiar with the WNCS, but have not explored it, 3; I’m familiar with the WNCS and have some experience with it, 4; I’m well acquainted with and use the WNCS

In addition to the strong facilitating effect of the normative belief of the manager, the normative beliefs of care provider colleagues were also identified as facilitators (D15A). Ninety-two percent of the care providers were motivated to comply with the opinions of the manager and healthcare worker colleagues, and 91% with the opinions of nurse colleagues (D15B). In all, 90% of the care providers reported that almost all colleagues used the WNCS as intended (D14). Moreover, the social support from the manager and nurse colleagues were described as facilitators by 80% of the care providers (D13).

The self-efficacy determinant (D16) encompassed several strong facilitating items. Three were related to the smart phone carried by all care providers during their watch. The majority of the care providers were confident that they could receive (96%) and manage (91%) alarms using the smart phone, and use the emergency call application in order to alert a colleague (90%). Eighty-two percent felt confident that they could provide feedback regarding the WNCS to their manager or a super user. In all, 86% of the care providers knew enough to use the call system (D17) and 86% was aware of the content of the WNCS (D18). The majority of the care providers (84%) found the use of WNCS to be within their responsibility as a professional (D10).

The relative low score regarding the need for training and supervision on a regular basis indicated that the care providers had integrated the WNCS into their workflows, and was identified as a proxy marker for facilitation of the implementation (D17h, Table 8).

**Table 8:** Barriers to WNCS implementation

Determinant / *item*	Mean	SD	Median	Range	N	TD/D (%)	A/TA (%)
D8 Personal benefits							
*a) WNCS makes my work performance better*^	3.45	(1.11)	4	(1–5)	98	20	55
D8 Personal drawbacks*							
*WNCS is too demanding to learn*^1^	2.86	(1.13)	3	(5-1)	98	44	37
D16 Self-efficacy							
*g) I can operate the WNCS software on the PC*~	3.19	(1.40)	3	(1–5)	75	22	38
D17 Knowledge							
*b) I had sufficient prior knowledge when WNCS was introduced*^	3.04	(1.20)	3	(1–5)	98	37	37
*e) The mobile transceiver was demonstrated during training*^	3.81	(1.36)	4	(1–50)	98	23	70
*h) I need more training and supervision about WNCS*^	3.24	(1.14)	3	(1–5)	98	26	48
D24 Material resources and facilities^	3.67	(1.00)	4	(1–50	97	21	65

Abbreviations: *WNCS* wirelessnurse call system, *TD/D* totally disagree/disagree, *A/TA* agree/totally agree. TD/D value ≥20% indicates that the determinant or item is a barrier to the implementation. The percentage values are rounded to the nearest whole number. ^1^ In the personal drawback item, the barrier is expressed in the A/TA column. Response scales: ^ 1; totally disagree, 2; disagree, 3; neither agree nor disagree, 4; agree, 5; totally agree. * 1; totally agree, 2; agree, 3; neither agree nor disagree, 4; disagree, 5; totally disagree. ~ 1; most definitely not, 2; definitely not, 3; perhaps, perhaps not, 4; definitely, 5; most definitely

#### Barriers

Determinants and items to which ≥20% of responding care providers responded ‘totally disagree/disagree’ were identified as barriers and are presented in Table 8. The two greatest barriers were the care providers’ insufficient prior knowledge at the start of implementation (D17) and the difficulty to learn the WNCS (D8), reported by 37% of all the care providers. On the other hand 37% found their prior knowledge sufficient (D17), and 44% of the care providers did not find it demanding to learn the WNCS (D8). Twenty percent did not find their work to improve with the WNCS (D8), which also constitute a barrier.

Regarding self-efficacy (D16), 22% could not operate the WNCS software on a PC. The care providers were expected to instruct residents to use a mobile transceiver and 70% of the care providers had been provided with demonstrations of the mobile transceiver during training, nevertheless 22% had not (D16). Moreover, 21% did not perceive that they had been supplied with sufficient material resources (D24).

#### Overall comments

To sum up the results, a thorough mapping of the WNCS components and features, as well as the implementation strategies, allowed us to adapt the MIDI questionnaire to the WNCS implementations. The care providers’ responses identified 22 facilitators related to nine determinants, and six barriers related to five determinants. All the facilitators and most of the barriers were related to the adopting user, and one barrier was related to organization. No features of the WNCS were identified as barriers, nor was the complexity of use.

## Discussion

This is the first study to explore facilitators and barriers during the full-scale implementation of WNCSs in residential care facilities using the MIDI questionnaire. The care providers’ evaluation of the implementation identified far more facilitators than barriers. The most pronounced facilitators were identified by virtually all the care providers. The first were the expected outcomes of the WNCSs, the importance and probability of achieving shorter response time to calls and increased safety for residents and families. The second was the subjective norm, the perceived behavioural expectations, imposed on the care providers by the manager. The two greatest barriers were the care providers’ status of knowledge at the start of implementation and the difficulty to learn the WNCS. Overall, the item scores indicated that the WNCSs were well received and that the implementation strategies and processes were satisfactory. This was supported by the facilitating effects of the care providers having gained some experience with the systems, that they considered the WNCSs to be in line with their professional responsibilities, and that almost all of their colleagues used the systems as intended.

### Safety first

The expected and perceived facilitating effects of the WNCS outcomes related to enhanced safety, were in line with previous implementations of less advanced call systems in residential care [52]. Importantly, the WNCSs were perceived as safe, not just expected to be safe. This indicated that the ethical implications of the WNCS’ design and functions corresponded well with moral values of the care providers, as found by Detweiler and Hindriks [14] and Ienca, Wangmo, Jotterand, Kressig, and Elger [53]. Strong leadership combined with shared mental models among nursing staff have previously been found to be associated with prompt response to calls in hospital settings [4, 54], as well as in a geriatric evaluation facility [55]. Cappelen, Harris, Storm and Aase [56] found engaged nursing managers to be role models for promoting improvements to patient safety in Norwegian nursing homes. The role taken by unit managers in combination with the safety propositions of the WNCSs found in our study, indicates that patient safety probably will be safeguarded through the use of the new system.

### Motivating managers

The managers’ level of engagement and active involvement in the implementation of WNCSs appears to be higher in this study than in previous studies of health information technology implementation in residential care facilities, which reported a lack of involvement as well as lack of systematic planning and decision-making from managers [29, 31, 32, 35]. The care providers’ evaluation of the managers’ efforts supported the effect of an implementation strategy adopted by all care facilities; that the unit managers had learned to use the WNCS and taken an essential role for driving the implementation [57]. The importance of their role as implementation champions is also supported by Shea and Belden [58] who found the champions to impact the implementation process, the usage behaviour and the overall success of the specific technology. Moreover, a transformational leadership style, formulating a vision for the future and building nursing staff capacities, have been found to result in higher levels of success in implementing change initiatives in residential care facilities [59]. However, the determinants and moderators of middle managers’ role have not been explained [39]. Our study did not aim to investigate managers’ motivation, but the choice of implementation strategies as well as the results of the survey indicate that the managers were motivated. However, it is not conclusive as to whether the full-scale scope of the implementation involving all residents and nursing staff, a general increased interest in digital transformation, or perceived regulatory requirements as reported by Bezboruah, Paulson, and Smith [29], motivated the managers to be a driving force in the implementation process.

### Rapid competence building

The two most prominent barriers occurred at the outset of the implementation and were related to competence. This was not surprising, as there are discrepancies between care providers’ health technology proficiency as compared to the expectancies of the Norwegian government [60]. Competence was evaluated with respect to knowledge, skills, learning strategies and implementation strategies. Even though the WNCSs were perceived as difficult to learn and the prior level of knowledge was somewhat low, the care providers rated themselves and their colleagues as competent users of the WNCS within the first year of implementation. The ability to acquire and maintain clinical competency is the result of both personal factors as well as contributing factors in the work environment [61]. Within the window of time from the outset of the implementation until the survey was undertaken, the care providers had gained experience from using the WNCS devices. Most of them had acquired skills and increased their knowledge about the WNCSs through structured training-sessions, which is a recommended implementation strategy [35, 52, 62]. They could easily understand instructions given by their manager and communicate about the WNCS and the implementation. Once training had been provided, there seemed to be less need for further instructions than previously reported from similar settings [29, 31]. Bearing in mind that a cross-sectional study can not establish causality [63], implementation strategies involving training and support most likely contributed to these outcomes and the rapid change in competence. Learning during implementations in residential care has been found to be a process of making connections between new knowledge and skills, and existing knowledge and practices [37]. In our study, the care providers’ smart phone application skills in fact facilitated the implementation. This was partly due to the learning strategies they applied, such as self-training and gaining experience from using the system over time. Personal knowledge and skills from using smart phones in their private lives probably further contributed to the rapid and successful uptake of smart phones and applications [64], since 95% of the Norwegian population (aged 9–79) have access to a smart phone [65].

### Full-scale implementation with tiny innovative steps

Although the mobile transceivers worn by the residents and the smartphones operated by the care providers represented new technology in the residential care settings, the tasks and routines implied by the WNCSs were much in line with the workflows known from previous call systems. The strategic decision initially to implement well known call system functionalities in full-scale and await the more complex functionalities was likely significant for the facilitating effects of the expected outcomes, ethical implications and competence building. Knowledge of and adherence to routines is fundamental to maintain patient safety [66], and the WNCSs were perceived not only to maintain, but to enhance patient safety. Such a connection between actions and outcomes has been found to further stimulate the learning process [37]. Thus, the organizational readiness seemed aligned with the challenges imposed during the implementation along the four dimensions proposed by Holt, Helfrich [67]: appropriateness, managerial support, self-efficacy and personal valence. In our study, the WNCS was perceived as appropriate for the RCF by the care providers; they found the managers to be supportive; they became confident about their self-efficacy; and, they found the WNCS personally beneficial. In contrast, technology implementations that simultaneously challenge care providers’ knowledge, values and workflows have been found to rely on resource intensive service innovations, compromising patient safety and predicting time consuming competence building and implementation processes [30, 31]. According to Bezboruah, Paulson, and Smith [29] most nursing homes do not realize the full potential benefits that implemented health IT systems offer. It remains unknown if the RCFs will utilize all the WNCS features procured.

### Implications for practice

This study has presented implementation strategies and WNCS functionalities, which seem to contribute to successful implementation, although not without complications. The importance of motivating managers was underscored, as was the impact of managers as role models with the ability to prepare the care unit for the implementation. The barriers identified in this study stress the urgency of providing equipment and material resources in due time, and offer training in the practical handling of the technology at the outset of the implementation. Nearly two out of five participants found it difficult to understand instructions provided by the vendors, which calls for specific attention to communication and information exchange between professions and groups involved in innovative implementations. This is in line with previous reports of differences in language and culture between technologists and care providers [32].

### Further research

For alarms to be effective, they must be part of a much more comprehensive care plan for each resident [9]. Some of the new digital functionalities offered by the WNCSs potentially expand and enrich the quality of care by allowing the care provider to remain focused on the residents, but may also have negative implications [1, 32, 68]. The more complex technologies that presumably disrupt established workflows and challenge existing patterns of interdependence among individuals or groups, will be more demanding to implement [69] and potentially pose new threats to patient safety. The ECRI institute [70] recently introduced missed alarms resulting from inappropriately configured secondary notification devices and systems on their Top 10 Health Technology Hazards, and further research on patient safety issues is needed as more of the novel WNCS-functionalities are introduced into clinical practice.

The care providers’ perceptions of the technology enhancing safety is likely to contribute to the residents’ feeling of increased safety [71]. This study did not include residents, and research on residents and families’ perspectives related to WNCSs should be undertaken.

### Strengths and limitations

This study contributes to the knowledge of full-scale implementation of WNCS. The research is however limited to the first phase of the full-scale implementation, as the RCFs implemented WNCS-functionalities that primarily supported established workflows and planned to implement new and more advanced functionalities over time.

The questionnaire applied took the perspective of the care provider, meaning that the perspectives of the administration and management, the support agencies, the vendors, as well as residents and families are not reported.

A large proportion (19.4%) of the respondents were WNCS super users, who had received more extensive training, which represents a bias.

The response rate to the questionnaire was low, which may have given a bias of the measures of outcome. Hence, we have not made comparisons between professional groups or the RCFs regarding the MIDI scores, but have reported from the entire group of participants. We do not know whether the characteristics of non-responders would differ from responders.

The current study was conducted within the first year of WNCS implementation. We were not able to investigate whether the time from the outset of implementation to the administration of the survey (e.g. 0–3 months, 4–6 months, 7–9 months, or 10–12 months) affected the results. Further, we were not able to contrast how the two conceptualisations of WNCS implementation, as an upgrade or as digital transformative processes, affected the implementations.

## Conclusions

The care providers gave an overall positive evaluation of the WNCS implementation. The expectations that the WNCS would lead to shorter response time and increased safety strongly facilitated the implementation, as did the firm influence and support by the manager and healthcare professionals within the residential care unit. Implementation barriers related to low levels of prior knowledge and perceived complexity inhibited adoption. The barriers seemed to be addressed by training and practicing technological skills. Further research is needed as more advanced WNCS functionalities are integrated into the residential care service. The MIDI questionnaire could be used for this purpose, with the inclusion of items adapted to the more advanced WNCS functionalities.

## Supplementary information

**Additional file 1 **MIDI questionnaire adapted to the wireless nurse call system (WNCS); translated from Norwegian

**Additional file 2.** MIDI determinants, including adopted items and participants’ response scores

## Abbreviations

A/TA: Agree/totally agree
D: Determinant
IoT: Internet of things
IT: Information technology
MIDI: Measurement Instrument for Determinants of Innovations
NCS: Nurse call system
RCF: Residential care facility
TD/D: Totally disagree/disagree
WNCS: Wireless nurse call system
