# Supplementary material for: Nursing staff’s evaluation of facilitators and barriers during implementation of wireless nurse call systems in residential care facilities. A cross-sectional study
Source: BMC Health Serv Res. 2020 Mar 4;20:163. doi: 10.1186/s12913-020-4998-9 (PMC7057572; doi:10.1186/s12913-020-4998-9)
Supplement: Supplementary file 1 — Additional file 1 MIDI questionnaire adapted to the wireless nurse call system (WNCS); translated from Norwegian [file 12913_2020_4998_MOESM1_ESM.docx]

**MIDI QUESTIONNAIRE ADAPTED TO WIRELESS NURSE CALL SYSTEM (WNCS);** *translated from Norwegian*

Profession: __________________________­­__ Position: ________________________________

Years of work experience: ____________ Years in current position: ___________________

Super user of WNCS? Yes No Gender: ______________ Age: ____________

**Please tick appropriate box**

| 1. WNCS clearly describes all activities and their order  \|  \| 1 \| totally disagree \| \| --- \| --- \| --- \| \|  \|  \|  \| \|  \| 2 \| disagree \| \|  \|  \|  \| \|  \| 3 \| neither agree nor disagree \| \|  \|  \|  \| \|  \| 4 \| agree \| \|  \|  \|  \| \|  \| 5 \| totally agree \| | 1. WNCS is based on factually correct knowledge  \|  \| 1 \| totally disagree \| \| --- \| --- \| --- \| \|  \|  \|  \| \|  \| 2 \| disagree \| \|  \|  \|  \| \|  \| 3 \| neither agree nor disagree \| \|  \|  \|  \| \|  \| 4 \| agree \| \|  \|  \|  \| \|  \| 5 \| totally agree \| |
| --- | --- | --- | --- | --- | --- | --- | --- | --- | --- | --- | --- | --- | --- | --- | --- | --- | --- | --- | --- | --- | --- | --- | --- | --- | --- | --- | --- | --- | --- | --- | --- | --- | --- | --- | --- | --- | --- | --- | --- | --- | --- | --- | --- | --- | --- | --- | --- | --- | --- | --- | --- | --- | --- | --- | --- |
| 1. Information and materials provided by WNCS are complete  \|  \| 1 \| totally disagree \| \| --- \| --- \| --- \| \|  \|  \|  \| \|  \| 2 \| disagree \| \|  \|  \|  \| \|  \| 3 \| neither agree nor disagree \| \|  \|  \|  \| \|  \| 4 \| agree \| \|  \|  \|  \| \|  \| 5 \| totally agree \| | 1. WNCS is too complex for me to use  \|  \| 1 \| totally agree \| \| --- \| --- \| --- \| \|  \|  \|  \| \|  \| 2 \| agree \| \|  \|  \|  \| \|  \| 3 \| neither agree nor disagree \| \|  \|  \|  \| \|  \| 4 \| disagree \| \|  \|  \|  \| \|  \| 5 \| totally disagree \| |
|  |  |
| 1. WNCS is a good match for how I am used to working  \|  \| 1 \| totally disagree \| \| --- \| --- \| --- \| \|  \|  \|  \| \|  \| 2 \| disagree \| \|  \|  \|  \| \|  \| 3 \| neither agree nor disagree \| \|  \|  \|  \| \|  \| 4 \| agree \| \|  \|  \|  \| \|  \| 5 \| totally agree \| | 1. The outcomes of using WNCS are clearly observable  \|  \| 1 \| totally disagree \| \| --- \| --- \| --- \| \|  \|  \|  \| \|  \| 2 \| disagree \| \|  \|  \|  \| \|  \| 3 \| neither agree nor disagree \| \|  \|  \|  \| \|  \| 4 \| agree \| \|  \|  \|  \| \|  \| 5 \| totally agree \| |
| 1. I think WNCS is relevant for the residents  \|  \| 1 \| totally disagree \| \| --- \| --- \| --- \| \|  \|  \|  \| \|  \| 2 \| disagree \| \|  \|  \|  \| \|  \| 3 \| neither agree nor disagree \| \|  \|  \|  \| \|  \| 4 \| agree \| \|  \|  \|  \| \|  \| 5 \| totally agree \| | 1. WNCS makes my work performance better  \|  \| 1 \| totally disagree \| \| --- \| --- \| --- \| \|  \|  \|  \| \|  \| 2 \| disagree \| \|  \|  \|  \| \|  \| 3 \| neither agree nor disagree \| \|  \|  \|  \| \|  \| 4 \| agree \| \|  \|  \|  \| \|  \| 5 \| totally agree \| |
| 1. WNCS makes my work more efficient  \|  \| 1 \| totally disagree \| \| --- \| --- \| --- \| \|  \|  \|  \| \|  \| 2 \| disagree \| \|  \|  \|  \| \|  \| 3 \| neither agree nor disagree \| \|  \|  \|  \| \|  \| 4 \| agree \| \|  \|  \|  \| \|  \| 5 \| totally agree \| | 1. WNCS makes my work more interesting  \|  \| 1 \| totally disagree \| \| --- \| --- \| --- \| \|  \|  \|  \| \|  \| 2 \| disagree \| \|  \|  \|  \| \|  \| 3 \| neither agree nor disagree \| \|  \|  \|  \| \|  \| 4 \| agree \| \|  \|  \|  \| \|  \| 5 \| totally agree \| |
| 1. WNCS makes my work safer  \|  \| 1 \| totally disagree \| \| --- \| --- \| --- \| \|  \|  \|  \| \|  \| 2 \| disagree \| \|  \|  \|  \| \|  \| 3 \| neither agree nor disagree \| \|  \|  \|  \| \|  \| 4 \| agree \| \|  \|  \|  \| \|  \| 5 \| totally agree \| | 1. WNCS makes me better prepared for other technologies  \|  \| 1 \| totally disagree \| \| --- \| --- \| --- \| \|  \|  \|  \| \|  \| 2 \| disagree \| \|  \|  \|  \| \|  \| 3 \| neither agree nor disagree \| \|  \|  \|  \| \|  \| 4 \| agree \| \|  \|  \|  \| \|  \| 5 \| totally agree \| |
| 1. It is my responsibility as a professional to use WNCS  \|  \| 1 \| totally disagree \| \| --- \| --- \| --- \| \|  \|  \|  \| \|  \| 2 \| disagree \| \|  \|  \|  \| \|  \| 3 \| neither agree nor disagree \| \|  \|  \|  \| \|  \| 4 \| agree \| \|  \|  \|  \| \|  \| 5 \| totally agree \| | 1. WNCS implies more benefits than drawbacks to me  \|  \| 1 \| totally disagree \| \| --- \| --- \| --- \| \|  \|  \|  \| \|  \| 2 \| disagree \| \|  \|  \|  \| \|  \| 3 \| neither agree nor disagree \| \|  \|  \|  \| \|  \| 4 \| agree \| \|  \|  \|  \| \|  \| 5 \| totally agree \| |
| 1. WNCS is too demanding to learn  \|  \| 1 \| totally agree \| \| --- \| --- \| --- \| \|  \|  \|  \| \|  \| 2 \| agree \| \|  \|  \|  \| \|  \| 3 \| neither agree nor disagree \| \|  \|  \|  \| \|  \| 4 \| disagree \| \|  \|  \|  \| \|  \| 5 \| totally disagree \| | 1. It is important that WNCS increases safety for residents  \|  \| 1 \| totally disagree \| \| --- \| --- \| --- \| \|  \|  \|  \| \|  \| 2 \| disagree \| \|  \|  \|  \| \|  \| 3 \| neither agree nor disagree \| \|  \|  \|  \| \|  \| 4 \| agree \| \|  \|  \|  \| \|  \| 5 \| totally agree \| |
| 1. It is probable that WNCS increases safety for residents  \|  \| 1 \| most definitely not \| \| --- \| --- \| --- \| \|  \|  \|  \| \|  \| 2 \| definitely not \| \|  \|  \|  \| \|  \| 3 \| perhaps, perhaps not \| \|  \|  \|  \| \|  \| 4 \| definitely \| \|  \|  \|  \| \|  \| 5 \| most definitely \| | 1. It is important that WNCS gives faster assistance to residents  \|  \| 1 \| totally disagree \| \| --- \| --- \| --- \| \|  \|  \|  \| \|  \| 2 \| disagree \| \|  \|  \|  \| \|  \| 3 \| neither agree nor disagree \| \|  \|  \|  \| \|  \| 4 \| agree \| \|  \|  \|  \| \|  \| 5 \| totally agree \| |
| 1. It is probable that WNCS gives faster assistance to residents  \|  \| 1 \| most definitely not \| \| --- \| --- \| --- \| \|  \|  \|  \| \|  \| 2 \| definitely not \| \|  \|  \|  \| \|  \| 3 \| perhaps, perhaps not \| \|  \|  \|  \| \|  \| 4 \| definitely \| \|  \|  \|  \| \|  \| 5 \| most definitely \| | 1. It is important that WNCS increases safety for families  \|  \| 1 \| totally disagree \| \| --- \| --- \| --- \| \|  \|  \|  \| \|  \| 2 \| disagree \| \|  \|  \|  \| \|  \| 3 \| neither agree nor disagree \| \|  \|  \|  \| \|  \| 4 \| agree \| \|  \|  \|  \| \|  \| 5 \| totally agree \| |
| 1. It is probable that WNCS increases safety for families  \|  \| 1 \| most definitely not \| \| --- \| --- \| --- \| \|  \|  \|  \| \|  \| 2 \| definitely not \| \|  \|  \|  \| \|  \| 3 \| perhaps, perhaps not \| \|  \|  \|  \| \|  \| 4 \| definitely \| \|  \|  \|  \| \|  \| 5 \| most definitely \| | 1. Residents will be satisfied when I use WNCS  \|  \| 1 \| totally disagree \| \| --- \| --- \| --- \| \|  \|  \|  \| \|  \| 2 \| disagree \| \|  \|  \|  \| \|  \| 3 \| neither agree nor disagree \| \|  \|  \|  \| \|  \| 4 \| agree \| \|  \|  \|  \| \|  \| 5 \| totally agree \| |
| 1. Residents will cooperate when I use WNCS  \|  \| 1 \| totally disagree \| \| --- \| --- \| --- \| \|  \|  \|  \| \|  \| 2 \| disagree \| \|  \|  \|  \| \|  \| 3 \| neither agree nor disagree \| \|  \|  \|  \| \|  \| 4 \| agree \| \|  \|  \|  \| \|  \| 5 \| totally agree \| | 1. Families will be satisfied when I use WNCS  \|  \| 1 \| totally disagree \| \| --- \| --- \| --- \| \|  \|  \|  \| \|  \| 2 \| disagree \| \|  \|  \|  \| \|  \| 3 \| neither agree nor disagree \| \|  \|  \|  \| \|  \| 4 \| agree \| \|  \|  \|  \| \|  \| 5 \| totally agree \| |
| 1. Families will cooperate when I use WNCS  \|  \| 1 \| totally disagree \| \| --- \| --- \| --- \| \|  \|  \|  \| \|  \| 2 \| disagree \| \|  \|  \|  \| \|  \| 3 \| neither agree nor disagree \| \|  \|  \|  \| \|  \| 4 \| agree \| \|  \|  \|  \| \|  \| 5 \| totally agree \| | 1. The proportion of my colleagues that use WNCS as intended  \|  \| 1 \| not a single colleague \| \| --- \| --- \| --- \| \|  \|  \|  \| \|  \| 2 \| almost no colleagues \| \|  \|  \|  \| \|  \| 3 \| a minority \| \|  \|  \|  \| \|  \| 4 \| half \| \|  \|  \|  \| \|  \| 5 \| a majority \| \|  \|  \|  \| \|  \| 6 \| almost all colleagues \| \|  \|  \|  \| \|  \| 7 \| all colleagues \| |

1. To use WNCS, I can get support from (please tick appropriate box for each person/function)

|  | | totally disagree | disagree | neither agree nor disagree | agree | totally agree |
| --- | --- | --- | --- | --- | --- | --- |
| a | the manager |  |  |  |  |  |
| b | a super user |  |  |  |  |  |
| c | a union representative |  |  |  |  |  |
| d | a nurse |  |  |  |  |  |
| e | a healthcare worker |  |  |  |  |  |
| f | the IT-service |  |  |  |  |  |
| g | the janitor |  |  |  |  |  |
| h | the vendors |  |  |  |  |  |

1. To what degree do the following persons expect you to use WNCS?

(Please tick appropriate box for each person/function)

|  | | most definitely not | definitely not | perhaps, perhaps not | definitely | most definitely |
| --- | --- | --- | --- | --- | --- | --- |
| a | the manager |  |  |  |  |  |
| b | a super user |  |  |  |  |  |
| c | a union representative |  |  |  |  |  |
| d | a nurse |  |  |  |  |  |
| e | a healthcare worker |  |  |  |  |  |
| f | the IT-service |  |  |  |  |  |
| g | the janitor |  |  |  |  |  |
| h | the vendors |  |  |  |  |  |
| i | the residents |  |  |  |  |  |
| j | the families |  |  |  |  |  |

1. When it comes to working with WNCS, to what extent do you comply with the opinions of the following persons? (Please tick appropriate box for each person/function)

|  | | very little | little | not a little, not a lot | a lot | a great deal |
| --- | --- | --- | --- | --- | --- | --- |
| a | the manager |  |  |  |  |  |
| b | a super user |  |  |  |  |  |
| c | a union representative |  |  |  |  |  |
| d | a nurse |  |  |  |  |  |
| e | a healthcare worker |  |  |  |  |  |
| f | the IT-service |  |  |  |  |  |
| g | the janitor |  |  |  |  |  |
| h | the vendors |  |  |  |  |  |
| i | the residents |  |  |  |  |  |
| j | the families |  |  |  |  |  |

1. To what extent are you familiar with WNCS?

|  | 1 | I’m not familiar with the WNCS |
| --- | --- | --- |
|  | 2 |  |
|  | 2 | I’m familiar with the WNCS, but have not explored it |
|  | 43 |  |
|  | 3 | I’m familiar with the WNCS and have some experience with it |
|  |  |  |
|  | 4 | I’m well acquainted with and use the WNCS |

| 1. I know enough to use WNCS  \|  \| 1 \| totally disagree \| \| --- \| --- \| --- \| \|  \|  \|  \| \|  \| 2 \| disagree \| \|  \|  \|  \| \|  \| 3 \| neither agree nor disagree \| \|  \|  \|  \| \|  \| 4 \| agree \| \|  \|  \|  \| \|  \| 5 \| totally agree \| | 1. I had sufficient prior knowledge when WNCS was introduced  \|  \| 1 \| totally disagree \| \| --- \| --- \| --- \| \|  \|  \|  \| \|  \| 2 \| disagree \| \|  \|  \|  \| \|  \| 3 \| neither agree nor disagree \| \|  \|  \|  \| \|  \| 4 \| agree \| \|  \|  \|  \| \|  \| 5 \| totally agree \| |
| --- | --- | --- | --- | --- | --- | --- | --- | --- | --- | --- | --- | --- | --- | --- | --- | --- | --- | --- | --- | --- | --- | --- | --- | --- | --- | --- | --- | --- | --- | --- | --- | --- | --- | --- | --- | --- | --- | --- | --- | --- | --- | --- | --- | --- | --- | --- | --- | --- | --- | --- | --- | --- | --- | --- | --- |
| 1. I was offered training before I started using WNCS  \|  \| 1 \| totally disagree \| \| --- \| --- \| --- \| \|  \|  \|  \| \|  \| 2 \| disagree \| \|  \|  \|  \| \|  \| 3 \| neither agree nor disagree \| \|  \|  \|  \| \|  \| 4 \| agree \| \|  \|  \|  \| \|  \| 5 \| totally agree \| | 1. I have participated in training-sessions  \|  \| 1 \| totally disagree \| \| --- \| --- \| --- \| \|  \|  \|  \| \|  \| 2 \| disagree \| \|  \|  \|  \| \|  \| 3 \| neither agree nor disagree \| \|  \|  \|  \| \|  \| 4 \| agree \| \|  \|  \|  \| \|  \| 5 \| totally agree \| |
| 1. The mobile transceiver was demonstrated during training  \|  \| 1 \| totally disagree \| \| --- \| --- \| --- \| \|  \|  \|  \| \|  \| 2 \| disagree \| \|  \|  \|  \| \|  \| 3 \| neither agree nor disagree \| \|  \|  \|  \| \|  \| 4 \| agree \| \|  \|  \|  \| \|  \| 5 \| totally agree \| | 1. The smart phone was demonstrated during training  \|  \| 1 \| totally disagree \| \| --- \| --- \| --- \| \|  \|  \|  \| \|  \| 2 \| disagree \| \|  \|  \|  \| \|  \| 3 \| neither agree nor disagree \| \|  \|  \|  \| \|  \| 4 \| agree \| \|  \|  \|  \| \|  \| 5 \| totally agree \| |
| 1. I have practiced using WNCS applications during idle time  \|  \| 1 \| totally disagree \| \| --- \| --- \| --- \| \|  \|  \|  \| \|  \| 2 \| disagree \| \|  \|  \|  \| \|  \| 3 \| neither agree nor disagree \| \|  \|  \|  \| \|  \| 4 \| agree \| \|  \|  \|  \| \|  \| 5 \| totally agree \| | 1. I need more training and supervision about WNCS  \|  \| 1 \| totally disagree \| \| --- \| --- \| --- \| \|  \|  \|  \| \|  \| 2 \| disagree \| \|  \|  \|  \| \|  \| 3 \| neither agree nor disagree \| \|  \|  \|  \| \|  \| 4 \| agree \| \|  \|  \|  \| \|  \| 5 \| totally agree \| |
| 1. I understand instructions provided by super users  \|  \| 1 \| totally disagree \| \| --- \| --- \| --- \| \|  \|  \|  \| \|  \| 2 \| disagree \| \|  \|  \|  \| \|  \| 3 \| neither agree nor disagree \| \|  \|  \|  \| \|  \| 4 \| agree \| \|  \|  \|  \| \|  \| 5 \| totally agree \| | 1. I understand instructions provided by the manager  \|  \| 1 \| totally disagree \| \| --- \| --- \| --- \| \|  \|  \|  \| \|  \| 2 \| disagree \| \|  \|  \|  \| \|  \| 3 \| neither agree nor disagree \| \|  \|  \|  \| \|  \| 4 \| agree \| \|  \|  \|  \| \|  \| 5 \| totally agree \| |
| 1. I understand instructions provided by the vendors  \|  \| 1 \| totally disagree \| \| --- \| --- \| --- \| \|  \|  \|  \| \|  \| 2 \| disagree \| \|  \|  \|  \| \|  \| 3 \| neither agree nor disagree \| \|  \|  \|  \| \|  \| 4 \| agree \| \|  \|  \|  \| \|  \| 5 \| totally agree \| | 1. I need to discuss WNCS challenges and experiences  \|  \| 1 \| totally disagree \| \| --- \| --- \| --- \| \|  \|  \|  \| \|  \| 2 \| disagree \| \|  \|  \|  \| \|  \| 3 \| neither agree nor disagree \| \|  \|  \|  \| \|  \| 4 \| agree \| \|  \|  \|  \| \|  \| 5 \| totally agree \| |
|  | |

1. Should you wish to do so, do you think you can …? (Please tick appropriate box for each activity)

|  | | most definitely not | definitely not | perhaps, perhaps not | definitely | most definitely |
| --- | --- | --- | --- | --- | --- | --- |
| a | teach a resident to use the mobile transceiver |  |  |  |  |  |
| b | instruct and answer questions from families |  |  |  |  |  |
| c | find a solution if the mobile transceiver doesn’t work |  |  |  |  |  |
| d | receive an alarm on the smart phone |  |  |  |  |  |
| e | manage an alarm on the smart phone |  |  |  |  |  |
| f | use the emergency call application to alert a colleague |  |  |  |  |  |
| g | operate the WNCS software on the PC |  |  |  |  |  |
| h | provide feedback to the manager or a super user |  |  |  |  |  |
| i | find information about the WNCS |  |  |  |  |  |
| j | participate in training sessions |  |  |  |  |  |

Please tick appropriate box

|  | | | yes | | no | I don’t know | |  |  |
| --- | --- | --- | --- | --- | --- | --- | --- | --- | --- |
|  | Has the management set up formal arrangements relating to the use of WNCS (in policy plans, work plans and so on)? | |  | |  |  | |  |  |
|  | A manager/super user is responsible for WNCS implementation | |  | |  |  | |  |  |
|  | The IT service bears responsibility in WNCS implementation | |  | |  |  | |  |  |
|  | Are there, in addition to the WNCS implementation, any other changes in the organisation affecting the implementation of the innovation now or in the foreseeable future (reorganisation, merger, cuts, staffing changes, other innovations)? | |  | |  |  | |  |  |
|  | | | |  | | |  |  |  |
| 1. New colleagues are prepared to use WNCS  \|  \| 1 \| totally disagree \| \| --- \| --- \| --- \| \|  \|  \|  \| \|  \| 2 \| disagree \| \|  \|  \|  \| \|  \| 3 \| neither agree nor disagree \| \|  \|  \|  \| \|  \| 4 \| agree \| \|  \|  \|  \| \|  \| 5 \| totally agree \| | | | 1. We are enough people to use WNCS as intended      \|  \| 1 \| totally disagree \| \| --- \| --- \| --- \| \|  \|  \|  \| \|  \| 2 \| disagree \| \|  \|  \|  \| \|  \| 3 \| neither agree nor disagree \| \|  \|  \|  \| \|  \| 4 \| agree \| \|  \|  \|  \| \|  \| 5 \| totally agree \| | | | | | | |

| 1. There are enough financial resources available to use the WNCS as intended  \|  \| 1 \| totally disagree \| \| --- \| --- \| --- \| \|  \|  \|  \| \|  \| 2 \| disagree \| \|  \|  \|  \| \|  \| 3 \| neither agree nor disagree \| \|  \|  \|  \| \|  \| 4 \| agree \| \|  \|  \|  \| \|  \| 5 \| totally agree \| | 1. I have been provided with enough time to include WNCS as intended in my day-to-day work  \|  \| 1 \| totally disagree \| \| --- \| --- \| --- \| \|  \|  \|  \| \|  \| 2 \| disagree \| \|  \|  \|  \| \|  \| 3 \| neither agree nor disagree \| \|  \|  \|  \| \|  \| 4 \| agree \| \|  \|  \|  \| \|  \| 5 \| totally agree \| |
| --- | --- | --- | --- | --- | --- | --- | --- | --- | --- | --- | --- | --- | --- | --- | --- | --- | --- | --- | --- | --- | --- | --- | --- | --- | --- | --- | --- | --- | --- | --- | --- | --- | --- | --- | --- | --- | --- | --- | --- | --- | --- | --- | --- | --- | --- | --- | --- | --- | --- | --- | --- | --- | --- | --- | --- |
| 1. I have been provided with enough materials and other resources or facilities necessary for the use of WNCS as intended  \|  \| 1 \| totally disagree \| \| --- \| --- \| --- \| \|  \|  \|  \| \|  \| 2 \| disagree \| \|  \|  \|  \| \|  \| 3 \| neither agree nor disagree \| \|  \|  \|  \| \|  \| 4 \| agree \| \|  \|  \|  \| \|  \| 5 \| totally agree \| | 1. Feedback is regularly provided about progress with the implementation of WNCS  \|  \| 1 \| totally disagree \| \| --- \| --- \| --- \| \|  \|  \|  \| \|  \| 2 \| disagree \| \|  \|  \|  \| \|  \| 3 \| neither agree nor disagree \| \|  \|  \|  \| \|  \| 4 \| agree \| \|  \|  \|  \| \|  \| 5 \| totally agree \| |
| 1. The activities included in WNCS fall within existing legislation and regulations  \|  \| 1 \| totally disagree \| \| --- \| --- \| --- \| \|  \|  \|  \| \|  \| 2 \| disagree \| \|  \|  \|  \| \|  \| 3 \| neither agree nor disagree \| \|  \|  \|  \| \|  \| 4 \| agree \| \|  \|  \|  \| \|  \| 5 \| totally agree \| |  |
