# Supplementary material for: Nursing staff’s evaluation of facilitators and barriers during implementation of wireless nurse call systems in residential care facilities. A cross-sectional study
Source: BMC Health Serv Res. 2020 Mar 4;20:163. doi: 10.1186/s12913-020-4998-9 (PMC7057572; doi:10.1186/s12913-020-4998-9)
Supplement: Supplementary file 2 — Additional file 2. MIDI determinants, including adopted items and participants’ response scores [file 12913_2020_4998_MOESM2_ESM.docx]

**Additional file 1. MIDI determinants, including adopted items and participants’ response scores**

| Determinant | Item specification | Mean | SD | Median | Range | n | Totally disagree/  Disagree,  or No (%) | Agree/  Totally agree,  or Yes (%) |
| --- | --- | --- | --- | --- | --- | --- | --- | --- |
| **Innovation: WNCS** | |  |  |  |  |  |  |  |
| D1 Procedural clarity ^1^ | WNCS clearly describes all activities and their order | 3.81 | (0.89) | 4 | (1-5) | 96 | 9 | 73 |
| D2 Correctness ^1^ | WNCS is based on factually correct knowledge | 3.58 | (0.98) | 4 | (1-5) | 95 | 10 | 55 |
| D3 Completeness ^1^ | Information and materials provided by WNCS are complete | 3.56 | (0.88) | 4 | (2-5) | 97 | 13 | 57 |
| D4 Complexity ^2^ | WNCS is too complex for me to use | 3.91 | (0.96) | 4 | (5-1) | 97 | 77 | 8 |
| D5 Compatibility ^1^ | WNCS is a good match for how I am used to working | 3.78 | (0.93) | 4 | (1-5) | 98 | 7 | 69 |
| D6 Observability ^1^ | The outcomes of using WNCS are clearly observable | 3.70 | (0.83) | 4 | (2-5) | 98 | 5 | 61 |
| D7 Relevance for resident ^1^ | I think WNCS is relevant for the residents | 3.94 | (0.81) | 4 | (2-5) | 97 | 5 | 77 |
| **Adopting user: care provider** | |  |  |  |  |  |  |  |
| D8 Personal benefit ^1^ | a) WNCS makes my work performance better | 3.45 | (1.11) | 4 | (1-5) | 98 | 20 | 55 |
| D8 Personal benefit ^1^ | b) WNCS makes my work more efficient | 3.45 | (0.98) | 4 | (1-5) | 98 | 17 | 51 |
| D8 Personal benefit ^1^ | c) WNCS makes my work more interesting | 3.15 | (0.93) | 3 | (1-5) | 98 | 17 | 29 |
| D8 Personal benefit ^1^ | d) WNCS makes my work safer | 3.62 | (1.01) | 4 | (1-5) | 98 | 16 | 75 |
| D8 Personal benefit ^1^ | e) WNCS makes me better prepared for other technologies | 3.62 | (0.93) | 4 | (1-5) | 97 | 8 | 62 |
| D8 Personal benefit ^1^ | f) WNCS implies more benefits than drawbacks to me | 3.79 | (0.89) | 4 | (1-5) | 98 | 8 | 73 |
| D8 Personal drawback ^2^ | WNCS is too demanding to learn | 2.86 | (1.13) | 3 | (5-1) | 98 | 44 | 37 |
| D9 Outcome expectation ^1^ | a) It is important that WNCS increases safety for residents | 3.78 | (0.47) | 4 | (2-4) | 98 | 0 | 98 |
| D9 Outcome expectation ^3^ | b) It is probable that WNCS increases safety for residents | 4.63 | (0.61) | 5 | (1-5) | 98 | 1 | 98 |
| D9 Outcome expectation ^1^ | c) It is important that WNCS gives faster assistance to residents | 3.76 | (0.45) | 4 | (2-4) | 97 | 0 | 98 |
| D9 Outcome expectation ^3^ | d) It is probable that WNCS gives faster assistance to residents | 4.54 | (0.74) | 5 | (1-5) | 98 | 3 | 95 |
| D9 Outcome expectation ^1^ | e) It is important that WNCS increases safety for families | 3.67 | (0.51) | 4 | (2-4) | 98 | 0 | 98 |
| D9 Outcome expectation ^3^ | f) It is probable that WNCS increases safety for families | 4.56 | (0.54) | 5 | (3-5) | 98 | 0 | 98 |
| D10 Professional obligation ^1^ | It is my responsibility as a professional to use WNCS | 4.02 | (0.91) | 4 | (1-5) | 98 | 7 | 84 |
| D11 Resident satisfaction ^1^ | a) Residents will be satisfied when I use WNCS | 3.99 | (0.78) | 4 | (2-5) | 95 | 3 | 74 |
| D11 Family satisfaction ^1^ | b) Families will be satisfied when I use WNCS | 3.94 | (0.72) | 4 | (3-5) | 93 | 0 | 71 |
| D12 Resident cooperation ^1^ | a) Residents will cooperate when I use WNCS | 3.73 | (0.78) | 4 | (1-5) | 98 | 3 | 59 |
| D12 Family cooperation ^1^ | b) Families will cooperate when I use WNCS | 3.84 | (0.72) | 4 | (3-5) | 96 | 0 | 64 |
| D13 Social support ^1^ | a) To use WNCS, I can get support from the manager | 4.23 | (0.97) | 4.5 | (1-5) | 96 | 6 | 80 |
| D13 Social support ^1,13^ | b) To use WNCS, I can get support from a super user | 4.62 | (0.69) | 5 | (2-5) | 76 | 1 | 70 |
| D13 Social support ^1^ | c) To use WNCS, I can get support from a union representative | 3.76 | (1.17) | 4 | (1-5) | 89 | 9 | 53 |
| D13 Social support ^1^ | d) To use WNCS, I can get support from a nurse | 4.18 | (0.81) | 4 | (1-5) | 95 | 2 | 80 |
| D13 Social support ^1^ | e) To use WNCS, I can get support from a healthcare worker | 4.14 | (0.74) | 4 | (3-5) | 92 | 0 | 75 |
| D13 Social support ^1^ | f) To use WNCS, I can get support from the IT service | 3.52 | (1.08) | 3 | (1-5) | 89 | 11 | 28 |
| D13 Social support ^1^ | g) To use WNCS, I can get support from the janitor | 3.04 | (1.20) | 3 | (1-5) | 90 | 26 | 29 |
| D13 Social support ^1^ | h) To use WNCS, I can get support from the vendors | 3.83 | (1.03) | 4 | (1-5) | 90 | 6 | 56 |
| D14 Descriptive norm ^4^ | The proportion of my colleagues that use WNCS as intended | 5.99 | (1.05) | 6 | (3-7) | 96 | 5 | 90 |
| D15A Normative beliefs ^3^ | a) The manager expects me to use WNCS | 4.76 | (0.48) | 5 | (3-5) | 98 | 0 | 98 |
| D15A Normative beliefs ^3,13^ | b) A super user expects me to use WNCS | 4.81 | (0.43) | 5 | (3-5) | 75 | 0 | 76 |
| D15A Normative beliefs ^3^ | c) A union representative expects me to use WNCS | 4.40 | (0.89) | 5 | (1-5) | 92 | 3 | 78 |
| D15A Normative beliefs ^3^ | d) A nurse colleague expects me to use WNCS | 4.55 | (0.66) | 5 | (2-5) | 97 | 1 | 92 |
| D15A Normative beliefs ^3^ | e) A healthcare worker colleague expects me to use WNCS | 4.48 | (0.65) | 5 | (2-5) | 97 | 1 | 93 |
| D15A Normative beliefs ^3^ | f) The IT service expects me to use WNCS | 4.13 | (1.03) | 4 | (1-5) | 87 | 6 | 68 |
| D15A Normative beliefs ^3^ | g) The janitor expects me to use WNCS | 3.70 | (1.25) | 4 | (1-5) | 89 | 14 | 53 |
| D15A Normative beliefs ^3^ | h) The vendors expect me to use WNCS | 4.40 | (0.88) | 5 | (1-5) | 88 | 3 | 76 |
| D15A Normative beliefs ^3^ | i) The residents expect me to use WNCS | 4.29 | (1.07) | 5 | (1-5) | 93 | 6 | 76 |
| D15A Normative beliefs ^3^ | j) The families expect me to use WNCS | 4.31 | (1.13) | 5 | (1-5) | 94 | 7 | 79 |
| D15B Motivation to comply ^5^ | a) I comply with opinions of the manager | 4.52 | (0.68) | 5 | (2-5) | 96 | 2 | 92 |
| D15B Motivation to comply ^5,13^ | b) I comply with opinions of a super user | 4.65 | (0.51) | 5 | (3-5) | 72 | 1 | 73 |
| D15B Motivation to comply ^5^ | c) I comply with opinions of a union representative | 4.08 | (1.01) | 4 | (1-5) | 90 | 7 | 76 |
| D15B Motivation to comply ^5^ | d) I comply with opinions of a nurse colleague | 4.42 | (0.59) | 4 | (3-5) | 95 | 0 | 91 |
| D15B Motivation to comply ^5^ | e) I comply with opinions of a healthcare worker colleague | 4.40 | (0.57) | 4 | (3-5) | 94 | 0 | 92 |
| D15B Motivation to comply ^5^ | f) I comply with opinions of the IT service | 4.05 | (0.93) | 4 | (1-5) | 87 | 3 | 65 |
| D15B Motivation to comply ^5^ | g) I comply with opinions of the janitor | 3.68 | (1.10) | 4 | (1-5) | 88 | 11 | 55 |
| D15B Motivation to comply ^5^ | h) I comply with opinions of the vendors | 4.17 | (0.81) | 4 | (2-5) | 87 | 2 | 70 |
| D15B Motivation to comply ^5^ | i) I comply with opinions of the residents | 4.21 | (1.08) | 5 | (1-5) | 92 | 7 | 78 |
| D15B Motivation to comply ^5^ | j) I comply with opinions of the families | 4.14 | (1.08) | 4 | (1-5) | 92 | 7 | 76 |
| D16 Self-efficacy ^3^ | a) I can teach a resident the mobile transceiver | 4.32 | (0.86) | 5 | (1-5) | 90 | 3 | 76 |
| D16 Self-efficacy ^3^ | b) I can instruct and answer questions from families | 4.17 | (0.83) | 4 | (2-5) | 94 | 3 | 77 |
| D16 Self-efficacy ^3^ | c) I can find a solution if the mobile transceiver doesn’t work | 3.27 | (1.08) | 3 | (1-5) | 94 | 19 | 40 |
| D16 Self-efficacy ^3^ | d) I can receive an alarm on the smart phone | 4.56 | (0.58) | 5 | (3-5) | 98 | 0 | 96 |
| D16 Self-efficacy ^3^ | e) I can manage an alarm on the smart phone | 4.49 | (0.69) | 5 | (2-5) | 97 | 1 | 90 |
| D16 Self-efficacy ^3^ | f) I can use the emergency call application to alert a colleague | 4.46 | (0.79) | 5 | (1-5) | 98 | 3 | 91 |
| D16 Self-efficacy ^3,13^ | g) I can operate the WNCS software on the PC | 3.19 | (1.40) | 3 | (1-5) | 75 | 22 | 38 |
| D16 Self-efficacy ^3^ | h) I can provide feed-back to the manager or super user | 4.31 | (0.87) | 5 | (1-5) | 97 | 3 | 82 |
| D16 Self-efficacy ^3,13^ | i) I can find information about the WNCS | 3.86 | (0.89) | 4 | (1-5) | 72 | 4 | 40 |
| D16 Self-efficacy ^3,13^ | j) I can participate in training sessions | 4.28 | (0.83) | 4 | (1-5) | 75 | 2 | 65 |
| D17 Knowledge ^1^ | a) I know enough to use WNCS | 4.18 | (0.77) | 4 | (1-5) | 97 | 3 | 86 |
| D17 Knowledge ^1^ | b) I had sufficient prior knowledge when WNCS was introduced | 3.04 | (1.20) | 3 | (1-5) | 98 | 37 | 37 |
| D17 Knowledge ^1,13^ | c) I was offered training before I started using WNCS | 4.18 | (0.96) | 4 | (1-5) | 77 | 5 | 68 |
| D17 Knowledge ^1,13^ | d) I have participated in training-sessions | 4.08 | (1.13) | 4 | (1-5) | 78 | 9 | 63 |
| D17 Knowledge ^1^ | e) The mobile transceiver was demonstrated during training | 3.81 | (1.36) | 4 | (1-5) | 98 | 23 | 70 |
| D17 Knowledge ^1^ | f) The smart phone was demonstrated during training | 4.02 | (1.08) | 4 | (1-5) | 98 | 10 | 79 |
| D17 Knowledge ^1^ | g) I have practiced using WNCS applications during idle time | 4.01 | (0.90) | 4 | (1-5) | 97 | 6 | 76 |
| D17 Knowledge ^1^ | h) I need more training and supervision about WNCS | 3.24 | (1.14) | 3 | (1-5) | 98 | 26 | 48 |
| D17 Knowledge ^1,13^ | i) I understand instructions provided by super users | 3.99 | (0.79) | 4 | (2-5) | 76 | 2 | 57 |
| D17 Knowledge ^1^ | j) I understand instructions provided by the manager | 3.92 | (0.84) | 4 | (1-5) | 97 | 5 | 74 |
| D17 Knowledge ^1^ | k) I understand instructions provided by the vendors | 3.47 | (1.05) | 3 | (1-5) | 95 | 10 | 44 |
| D17 Knowledge ^1^ | l) I need to discuss WNCS challenges and experiences | 3.73 | (1.00) | 4 | (1-5) | 97 | 9 | 60 |
| D18 Awareness of content ^6^ | The extent to which I am familiar with WNCS | 3.25 | (0.75) | 3 | (1-4) | 97 | 12 | 87 |
| **Organization: residential care facility unit** | |  |  |  |  |  |  |  |
| D19 Formal ratification ^7,8^ | Use of WNCS is integrated in plans |  |  |  |  | 95 | 9 | 50 |
| D20 Staff turnover ^1^ | New colleagues are prepared to use WNCS | 3.50 | (1.02) | 4 | (1-5) | 86 | 10 | 46 |
| D21 Staff capacity ^1^ | We are enough people to use WNCS as intended | 3.73 | (0.95) | 4 | (1-5) | 93 | 5 | 62 |
| D22 Financial resources ^1^ | WNCS is supported by sufficient financial resources | 3.40 | (0.88) | 3 | (1-5) | 88 | 8 | 48 |
| D23 Time available ^1^ | I have enough time available to use WNCS | 3.56 | (0.99) | 4 | (1-5) | 95 | 13 | 55 |
| D24 Material resources ^1^ | I have enough equipment to use WNCS | 3.67 | (1.00) | 4 | (1-5) | 97 | 21 | 65 |
| D25 Coordinator ^7, 9^ | Manager/super user is responsible for WNCS implementation |  |  |  |  | 95 | 2 | 61 |
| D25 Coordinator ^7, 10^ | The IT service bears responsibility in WNCS implementation |  |  |  |  | 95 | 11 | 36 |
| D26 Unsettled organization ^7,11^ | Major changes are ongoing in parallel to WNCS implementation |  |  |  |  | 90 | 9 | 35 |
| D27 WNCS use information ^1, 12^ | I can easily find information about WNCS use | 3.72 | (0.89) | 4 | (2-5) | 25 | 7 | 16 |
| D28 Performances feedback ^1^ | We get regular feedback about WNCS implementation | 3.29 | (0.94) | 3 | (1-5) | 98 | 17 | 39 |
| **Socio-political context: Norwegian legislation** | |  |  |  |  |  |  |  |
| D29 Legislation and regulations^1^ | WNCS activities fall within current regulations | 3.71 | (0.82) | 4 | (1-5) | 91 | 2 | 55 |

Abbreviations: D; determinant, WNCS; wireless nurse call system.

Response scales: ^1^ 1; totally disagree, 2; disagree, 3; neither agree nor disagree, 4; agree, 5; totally agree. ^2^ 1; totally agree, 2; agree, 3; neither agree nor disagree, 4; disagree, 5; totally disagree. ^3^ 1; most definitely not, 2; definitely not, 3; perhaps, perhaps not, 4; definitely, 5; most definitely. ^4^ 1; not a single colleague, 2; almost no colleagues, 3; a minority, 4; half, 5; a majority, 6; almost all colleagues, 7; all colleagues. ^5^ 1; very little, 2; little, 3; not a little, not a lot, 4; a lot, 5; a great deal. ^6^ 1; I’m not familiar with the WNCS, 2; I’m familiar with the WNCS, but have not explored it, 3; I’m familiar with the WNCS and have some experience with it, 4; I’m well acquainted with and use the WNCS. ^7^ 1; no, 2; yes, 3; I don’t know.

Other comments: ^8^ 38% replied I don’t know.  ^9^ 34% replied I don’t know. ^10^ 50% replied I don’t know. ^11^ 48% replied I don’t know. ^12^ only presented to RCF2. ^13^ not presented to RCF1.
